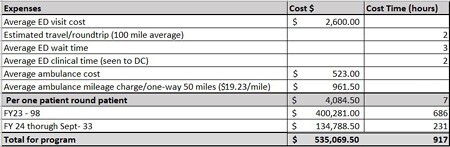# 561 Financial Savings of Utilizing TeleBurn for Burn Triage

**DOI:** 10.1093/jbcr/irae036.195

**Published:** 2024-04-17

**Authors:** Beth McGuire, Nicole P Bernal, Laura K Pezzopane

**Affiliations:** The Ohio State University Wexner Medical Center, Columbus, OH; The Ohio State University Wexner Medical Center, Columbus, OH; The Ohio State University Wexner Medical Center, Columbus, OH

## Abstract

**Introduction: Introduction:**

Hospital to hospital transfers through the emergency department (ED) after a burn injury for wound assessment/care followed by discharge is an unnecessary expense to the patient and the medical system. It presents waste to the healthcare system with EDs at capacity and having limited space/staffing.

Initially we used telemedicine stoke robot photography technology to connect the referring hospital with burn physicians through the transfer center. It has grown to include non-stroke network facilities using shared services via the electronic medical record. We sought to define the cost savings to the patient in time and dollars by expedited patient care, time reduction/resource utilization in patient flow, and show a net savings to the medical system and patient without additional time to the physicians.

**Methods: Methods:**

Regional outreach coordinators were given posters with the TeleBurn triage information for outlying facilities to educate providers how to initiate the process.

The Teleburn triage process starts with the transfer center nurses taking information/pictures when the referring hospital provider is asking for an opinion or possible follow-up. For small/non-emergent injuries, patients are scheduled in clinic within 48 hours; if they need surgery/admission, direct admission or outpatient procedures are arranged.

**Results: Results:**

Financials reviewed an average ED visit cost for time/travel. Over the course of the program there have been 133 patients that were triaged to clinic instead of the ED for a total cost saving of $535,069.50 and 917 hours of travel (Table 1). We continue to trend increased clinic patient visits and reduced ED visits with inpatient growth.

**Conclusions:**

Conclusion: TeleBurn triage process can be done in a HIPPA compliant manor without having to purchase new technology or require additional physician time. We demonstrated a significant cost/time/resource savings and improved the flow through the medical system.

**Applicability of Research to Practice: Applicability of Research to Practice:**

The TeleBurn triage program was not developed with the intention for billing these encounters but to expedite patient care and move the initial burn evaluation for smaller burns to the outpatient clinic from the ED. The program’s continued impact has led to improved connections with referring hospitals who are more confident with rapid connection and follow-up process.